# Dracunculiasis Eradication: End-Stage Challenges

**DOI:** 10.4269/ajtmh.22-0197

**Published:** 2022-06-27

**Authors:** Donald R. Hopkins, Adam J. Weiss, Fernando J. Torres-Velez, Sarah G. H. Sapp, Kashef Ijaz

**Affiliations:** ^1^ The Carter Center, Atlanta, Georgia;; ^2^ Centers for Disease Control and Prevention, Atlanta, Georgia

## Abstract

This report summarizes the status of the global Dracunculiasis Eradication Program as of the end of 2021. Dracunculiasis (Guinea worm disease) has been eliminated from 17 of 21 countries where it was endemic in 1986, when an estimated 3.5 million cases occurred worldwide. Only Chad, Ethiopia, Mali, and South Sudan reported cases in humans in 2021. Chad, Ethiopia, and Mali also reported indigenous infections of animals, mostly domestic dogs, with *Dracunculus medinensis*. Insecurity and infections in animals are the main obstacles remaining to interrupting dracunculiasis transmission completely.

## INTRODUCTION

Since the previous article in this series updated the program’s status as of 2017, when Guinea worm disease appeared to be limited to two of the four endemic countries and seemed on the verge of eradication,^1^ 4 years later the global Guinea Worm Eradication Program (GWEP) currently faces critical challenges in parts of five endemic countries. This article describes the status of the program as of the end of 2021.

A previous report described the parasite and the strategies and interventions then being used to eradicate it.^2^ Dracunculiasis (Guinea worm disease) is caused by the nematode parasite *Dracunculus medinensis*, and it is transmitted to humans in contaminated drinking water containing copepods (water fleas) that harbor infective larvae of the parasite. Recently the potential infection of humans by eating poorly cooked aquatic animals has emerged as a possible mode of infection.^3^ The larvae are expelled into water by adult female worms, and then, after humans consume copepods or aquatic animals with infective larvae, the adult worms emerge through the skin of infected persons about 1 year after infection. Once the end of the worm has emerged through the skin, the remainder of the worm, up to 1 meter long, must be removed. Emergence and removal of the worm is slow, painful, and often disabling with secondary infections, although usually not fatal, and therefore has a serious adverse socioeconomic impact on the health, agricultural productivity, and school attendance of affected populations. Without medical care, persons are incapacitated for periods averaging up to 3 months. In the past, more than one-half of a village’s population might have been affected simultaneously during the main harvest or planting season. Until significant numbers of infections with *D. medinensis* were discovered in dogs in Chad in 2012, humans were the only known reservoir of infection. Individual infections last only 1 year, but people do not develop immunity to the parasite.

There is no effective treatment or vaccine; however, the infection may be prevented by educating villagers about the origin of the disease, preventing infected persons from entering sources of drinking water, filtering all drinking water through a finely woven cloth that removes the copepods, applying Abate larvicide (temphos; BASF Corp., Florham Park, NJ) to kill the copepods in ponds or other stagnant sources of drinking water, and providing clean drinking water from safe sources, such as protected hand-dug or borehole wells. Transmission from a patient or infected animal is considered contained if the infection is detected before or within 24 hours of worm emergence; the patient or animal has not entered any water source since the worm emerged; a village volunteer or other health worker has properly managed the wound until the worm(s) is fully removed; the containment process, including verification that it is a case of Guinea worm disease, is verified by a supervisor; and Abate is used if there is any uncertainty about contamination of sources of drinking water or if such contamination is known to have occurred.

The global eradication campaign began at the CDC in 1980, it was adopted as a subgoal of the International Drinking Water Supply and Sanitation Decade (1981–1990), and it has been led since 1986 by The Carter Center, which is at the head of a coalition that includes the Ministries of Health of the endemic countries, the CDC, the WHO, and the United Nations Children’s Fund (UNICEF) as major partners and thousands of village volunteers and supervisory health staff. The coalition is supported by numerous donor agencies, governments, foundations, and other institutions. The 1991 World Health Assembly resolution WHA 44.5 set an eradication target date of 1995. When The Carter Center began leading the global campaign after the CDC, there were an estimated 3.5 million cases of dracunculiasis worldwide.^4^ At the World Health Assembly (WHA) in 2004, ministers of health set a new target to stop transmission of dracunculiasis by the end of 2009.^5^ When that target date was not met, partly because of the ongoing civil war in Sudan as well as unexpected outbreaks in Chad, Ethiopia, and Mali, the global initiative resolved to interrupt transmission as soon as possible. In a revised division of labor after major donors to the campaign reviewed the global program with staff from The Carter Center and WHO and other experts in 2014, The Carter Center assumed responsibility instead of WHO for assisting the remaining endemic countries to maintain surveillance in dracunculiasis-free areas, and nationwide for all 3 years after the last case instead of for only 1 year, while WHO continued to be responsible for certifying countries as dracunculiasis-free and for assisting them in the pre-certification stage. In 2021, the WHO launched its Roadmap for Neglected Tropical Diseases 2021–2030,^6^ which includes a target for eradicating Guinea worm by 2027 and certifying all Guinea worm-endemic countries as free of transmission by the end of 2030.

## CURRENT STATUS OF THE CAMPAIGN

**Figure 1.**
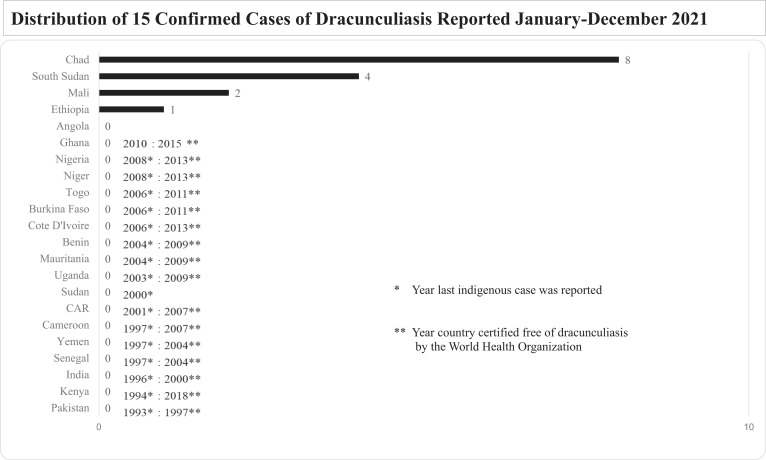
Distribution of 15 confirmed cases of dracunculiasis reported, January–December 2021.

At the end of 2021, only 15 human cases of dracunculiasis were reported from four countries (Chad, Ethiopia, Mali, South Sudan) worldwide (down from 26 cases in Angola, Chad, Ethiopia, and South Sudan in 2020, plus one imported case in Cameroon), in 14 villages (down from 18 villages with indigenous cases in 2020) (Figure 1). This is the fewest human Guinea worm cases reported globally for 1 year since the eradication program began. Chad, Ethiopia, and Mali also reported 863 animals, mostly dogs, with Guinea worm infections in 2021 (in addition to 10 imported dogs in Cameroon), compared with 1,102 infected animals in 2018 and 1,601 infected animals in 2020. The CDC has confirmed worms from all human cases, all infected animals outside of Chad, and a sample of worms from Chadian animals, as *D. medinensis* by microscopic and/or molecular examination.^7^

### Chad.

Chad rediscovered cases of dracunculiasis in humans in 2010 after having reported no cases since 2000.^8^ Genetic studies suggest that Guinea worms were still circulating in Chad during the period when Chad reported no cases.^9^ The Carter Center resumed assisting Chad’s national GWEP (CGWEP) in 2011 at the request of the Ministry of Health. The WHO declared Chad officially endemic again in 2012 after 3 consecutive years of indigenous cases in humans, and Chad discovered 27 domestic dogs (*Canis familiaris*) with Guinea worm infections for the first time that year. The CGWEP increased its villages under active village-based surveillance from 642 in 2011 to 1,860 in 2017, 2,332 in 2020, and 2,309 in 2021; it has publicized a cash reward equivalent to US$100 for reporting a human infection since before 2010 and US$20 for reporting an infected dog since 2015. According to spot checks of convenience samples in 2021, 87% of persons queried were aware of the cash reward. The program investigated 3,454 rumors of Guinea worm infections in humans and animals in 2017, 134,075 rumors in 2020, and 181,945 rumors in 2021. Chad and the WHO are paying special attention to disorder and disruption in Central African Republic and adjacent areas of Chad, and to prevention, containment, and detection of Guinea worm infections near Chad’s side of their shared border. During the period under review, Chad’s minister of health visited endemic villages in February 2019 and March 2020.

Guinea worm’s “peculiar epidemiology” in Chad,^3^ including its riverine ecology and association with annual mass fishing events along the Chari River in May–June at the end of the dry season, has persisted since the infection was rediscovered there in 2010. Except for a common-source outbreak of 22 cases in Bogam village in 2019,^10^ human Guinea worm infections in Chad occur sporadically with few small clusters of cases in families or villages in successive years and are vastly outnumbered by infections in dogs, which are highest in March–August and peak in June.^11^ Human cases in Chad peak in July–August, affect many different ethnicities, and two-thirds of the cases cluster in two of Chad’s 23 regions: Chari Baguirmi and Moyen Chari.^12^ The current hypothesis is that most humans and animals in Chad are infected by eating inadequately cooked or raw fish or other aquatic transport or paratenic hosts (hosts in which the larval parasite does not develop but remains viable, either transiently in the gut or more durably in the tissues, respectively).

Researchers have recovered *D. medinensis* L3 larvae from two types of wild frogs in Chad^13^ and from experimentally exposed feather fin catfish,^14^ which are common in the Chari River, and have shown in the laboratory that fish can serve as transport hosts for *Dracunculus* spp.^15^ and that *D. medinensis* can use frogs as paratenic hosts.^16^ They have also shown that viable *D. insignis* larvae can survive in tadpoles/frogs for 4 to 8 months in the laboratory.^14^ Other studies have associated increased risk of Guinea worm infections in Chadian dogs with consumption of fish,^17^ and consumption of raw fish guts (Eugene Liu, unpublished data). A small study by The Carter Center and Chadian staff of households with and without infected dogs suggests that dogs that accompanied their owners to mass fishing or were allowed to wander their village alone—both of which provide access to discarded fish or fish guts—are at greater risk of Guinea worm infection (Sarah Yerian, unpublished data). After an unsuccessful trial, the CGWEP and allied researchers began a second trial of flubendazole in dogs late in 2021, this time using a high-dose, single-encounter protocol to test the antihelminthic’s feasibility as a treatment to prevent development of Guinea worm infections in dogs.

The CGWEP began implementing enhanced health education in 2013 by urging villagers to cook their fish well and bury fish entrails. Spot inspections of sampled households found 74% practiced safe disposal of fish guts in 2015 and 81% or more after 2016. In 2014, the program began applying Abate in cordoned areas of large riverside lagoons and encouraging villagers to tether infected dogs until the worms were fully removed. It began more intense Abate applications to ponds in 70 communities with multiple infected dogs in 2017. The CGWEP expanded Abate coverage from 21% of 340 villages with a Guinea worm case or infection in 2018, to 92% of 444 villages in 2019, 100% of 436 villages in 2020, and 100% of 340 villages in 2021. The CGWEP tethered 40% of 113 infected dogs in 2014 and 76% of 817 infected dogs in 2017. Working with veterinarians and community leaders, in 2020 Chad began supporting proactive tethering of all dogs and cats in villages at risk during the peak transmission season. The minister of health announced the new proactive tethering strategy personally during a visit to an endemic village in March 2020. The program expanded the number of tethered dogs from a peak of 6,917 in September 2020 to 16,726 in September 2021, and the number of tethered cats from 225 in November 2020 to 6,125 a year later; it tethered 83% of 1,508 infected dogs in 2020 and 81% of 767 infected dogs in 2021. In 2021, villagers in one endemic village decided to give one-sixth of each household’s monthly US$20 equivalent allotment to support feeding and proactively tethering their animals, to a community fund for building and supporting a school, while keeping the rest for themselves. Other villages began adopting that innovative idea within 3 months. At the end of 2021 79% of 553 villages reporting infected animals or humans in 2020–2021 had at least one source of clean drinking water. The CGWEP included 1,855 staff and 11,986 village volunteers in 2021.

**Figure 2.**
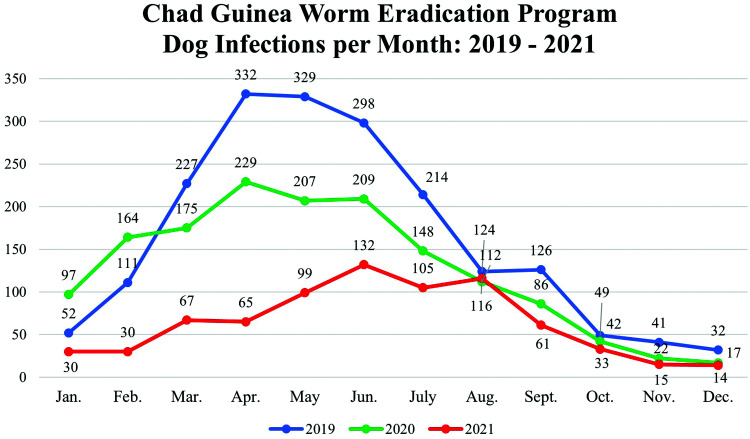
Chad Guinea Worm Eradication Program—dog infections per month, 2019–2021.

The number of reported infected dogs in Chad peaked at 1,935 in 2019, along with 61 cats. Following the scale-up of Abate coverage and proactive tethering of dogs in 2018–2020, Chad reduced dog infections by 20% (from 1,935 to 1,508) between 2019 and 2020 and by 49% (from 1,508 to 767) between 2020 and 2021 (Figure 2). Chad reduced its human Guinea worm cases, which peaked at 48 with a waterborne common-source outbreak in 2019 and averaged 14 cases (range: 8–26; omitting 22 cases in the common-source outbreak) in the years since 2010, by 75% (from 48 to 12) between 2019 and 2020 and by 33% from 2020 to 2021 (from 12 to 8). Chad contained 75% of its human cases and 82% of animal Guinea worm infections in 2021. The program identified the presumed source of two human infections in 2021; most animal infections occurred in villages that also reported Guinea worm infections in 2020 and probably were infected in their respective villages. Chad’s National Dracunculiasis Eradication Committee has been dormant for the past decade.

Cameroon reported one human case in 2019 and another in 2020 plus five dogs and a cat in 2020 and 10 dogs in 2021. The Guinea worm cases and animal infections in Cameroon in 2019–2021 have occurred in a cluster of communities with shared ethnicity, markets, and extended families of fishermen/farmers, some of whom live on both sides of the Logone River, which is the local border between Chad and Cameroon. Indigenous transmission was still occurring in Chad near that part of the border. So far, none of the cases or infected animals in Cameroon has been shown on investigation to have resided in Cameroon exclusively during the period when they likely acquired their Guinea worm infection.

### Ethiopia.

Ethiopia’s Dracunculiasis Eradication Program (EDEP) had 167 villages under active surveillance in 2017 in endemic Gog and Abobo districts of Gambella Region and adjacent Anfilo district of Oromia Region. This followed an outbreak of 15 cases in 2017 among seasonal workers from Anfilo who drank unfiltered water from a contaminated common source at a commercial farm in Abobo district the year before. It had 726 villages as well as 192 nonvillage areas under active surveillance in Gog and Abobo in 2021. The EDEP conducts extensive integrated surveys in cooperation with polio, trachoma, and other mass drug administration programs, reaching more than 150,000 persons that way in 2020 and 362,796 persons in 2021. In 2018, the program increased its cash reward for reporting a human case to the equivalent of US$360 (from $100) and for reporting an infected animal to US$40 (from $10). According to spot checks of convenience samples in 2021, 96% of persons queried in active surveillance areas were aware of the reward, and 14% or less were aware in nonendemic regions sampled. The EDEP investigated 13,433 rumors of Guinea worm infections in 2017 and 23,903 rumors in 2021. Ethiopia’s minister of health visited endemic areas in December 2018, February 2019, and December 2019.

Since 2012, endemic Guinea worm transmission in Ethiopia has been limited to a small, forested area of about 50 × 25 miles (80 × 40 km) in Gog district, where 60 dogs, 46 humans, 13 cats, and 20 wild olive baboons (*Papio anubis*) have been found to be infected during that time. Peak transmission occurs during the rainy season in April–August. Most cases were ethnic Agnuak. The current hypothesis is that except for three common-source waterborne outbreaks that occurred in or near villages, in recent years most Guinea worm infections among humans, dogs, and baboons in Ethiopia have occurred by drinking contaminated water from small ponds in the forest. The infected cats occurred in two small clusters in refugee camps. Of the 20 human cases in 2012–2016 (omitting common-source outbreaks in 2017 and 2020 and zero case years 2018–2019), 17 were males who averaged 39 years old (range 12–60 years), most of whom hunted, or gathered honey, wood, or other materials in the forest, often accompanied by dogs. Unlike Chad, a study of the ecology, ranging behavior, and diet of dogs in three villages of Gog district with infected dogs found no association between Guinea worm infection and dogs’ consumption of fish or frogs.^18^ The EDEP has worked with the Ethiopian Wildlife Conservation Authority, Ethiopian Public Health Institute, and The Carter Center, including veterinarians and other international partners since 2018 to support field teams and researchers in tracking and temporarily trapping, examining, and bleeding members of six baboon troops in the peridomestic endemic area. Those activities were partly disrupted in 2020 by the COVID-19 pandemic. They resumed in 2021 with the aim of assessing the extent of Guinea worm infection among wild baboons in the area but were suspended again because of COVID-19 and insecurity.

The EDEP has stressed health education and distributed cloth filters to villagers at risk since early in its campaign and in recent years promoted using pipe filters when drinking water in the forest, cooking fish thoroughly, and warning children against consuming raw “fingerlings” (small fish). About a quarter of at-risk villages and most commercial farms do not have a source of safe drinking water. By engaging hunters and using data from baboon trackers and others, the EDEP has identified and treated more and more ponds with Abate in forest areas associated with animal and human infections, advancing from 312 ponds treated in Gog district in July 2017 to 816 ponds treated there in July 2021, for example. The program plans to improve Abate coverage even further in 2022 by integrating data from ground tracking and GPS collars on baboons, with remote sensing and satellite imagery to help find and treat ponds used by baboons, including under dense forest canopy. The EDEP included 248 staff and 1,302 village volunteers in 2021.

**Figure 3.**
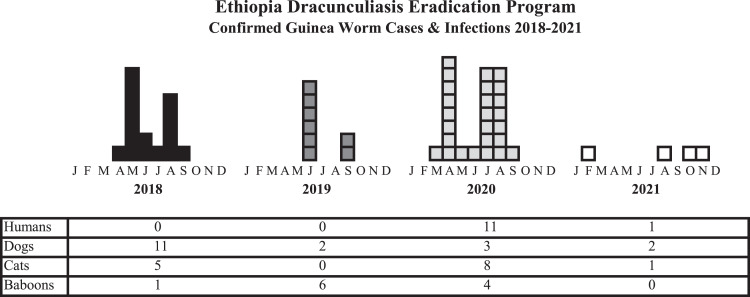
Ethiopia Dracunculiasis Eradication Program—confirmed guinea worm cases and infections, 2018–2021.

In April 2018, Ethiopian villagers and program staff decided that since dogs were still getting infected, perhaps tethering all dogs (and cats) in villages at risk during the peak transmission season would be more effective than only tethering dogs with emerging Guinea worms. In addition to containing infected dogs to prevent contamination of water sources as before, the novel approach of proactive tethering could also reduce all dogs’ exposure to potentially contaminated food and water as well as improve detection of dogs whose infections might otherwise have been missed or not detected in time to be contained. After introducing proactive tethering, including support for food, water, shelter, exercise, and veterinary care for the animals, dog infections in Ethiopia declined by 80% in 1 year, from an average 12.5 infections annually in 2015–2018 (range: 11–14) to two, three, and two infected dogs in 2019–2021, respectively (Figure 3). Ethiopia reported zero human cases for 27 consecutive months from January 2018 through March 2020 but had a common-source outbreak of 7 cases in April 2020 traced to a small water source used by humans and baboons that was inadvertently not treated soon after the rains began in 2019, and an outbreak of four cases later that year. Ethiopia reported one human case, two infected dogs, and one infected cat in 2021. The program reported zero infected baboons in 2021 for the first time in 9 years, despite villagers reporting that 81 baboons, which are treated as pests because they raid villagers’ crops, were killed, or found dead without evidence of Guinea worm infection in 14 villages in Gog district in April–August 2021. The EDEP contained all but one (a dog) of the four Guinea worm infections in 2021 and identified the presumed source (exposure to a known infected water body or visit to a location with a known Guinea worm infection 10 to 14 months before worm emergence) of all four infections. Ethiopia’s National Dracunculiasis Eradication Certification Committee inspected Guinea worm eradication activities in Gog and Abobo districts in 2019; it met twice in 2020 and three times in 2021.

### Mali.

Mali’s GWEP (MGWEP) has detected few human cases since an outbreak of 40 cases in 2014, and it found no human cases for 51 consecutive months from December 2015 through February 2020. Insecurity has constrained program activities in Gao, Kidal, Mopti, and Timbuktu Regions since a coup d’etat in 2012, but indigenous health workers trained by the program still conduct surveillance for Guinea worm and report to the MGWEP monthly in those areas. Mali had 455 villages under active surveillance in affected areas of the country in 2017, which it increased to 903 villages in 2018, 2,802 in 2019, 2,699 in 2020 and 2,216 villages in 2021. The program also conducts integrated surveys in cooperation with polio eradication activities, reaching 5,963 persons that way in 2020, for example. The MGWEP increased its cash reward for reporting a human Guinea worm case from the equivalent of US$100 since 2014 to US$340 in 2018 (dogs: US$20). Spot checks of convenience samples in areas under active surveillance found that approximately 77% were aware of the rewards for reporting infected humans and dogs in 2019, 89% in 2020, and 91% in 2021. The program received and investigated relatively few rumors of human and animal infections: 477 rumors in 2017, 192 in 2020, and 510 in 2021.

**Figure 4.**
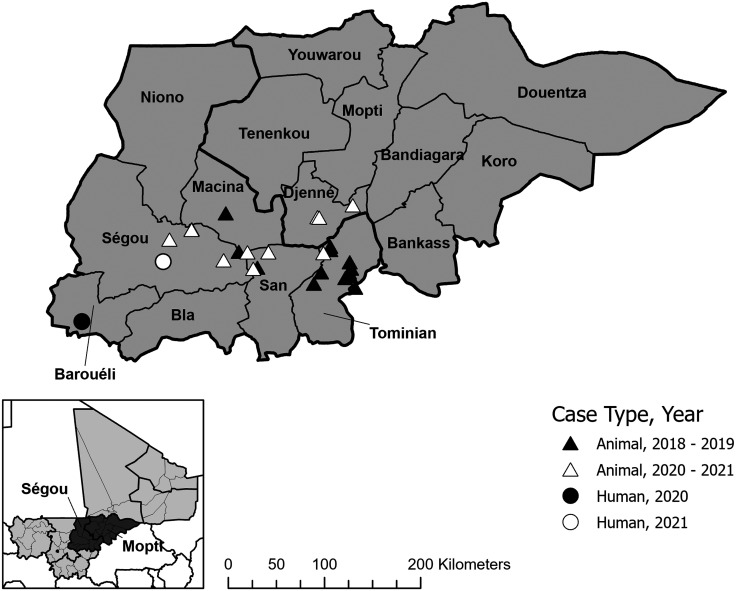
Map showing villages reporting guinea worm in humans and/or animals in Mali, 2018–2021.

Mali reported its first dog with a Guinea worm infection in 2015 and reported an average of 11.8 infected dogs per year (range: 8–18) in 2016–2021. It reported five infected cats in 2017–2021. The human and animal infections since 2015 have occurred in the inland delta of the Niger River, comprising parts of Mopti and Segou Regions in an ecological zone similar to Chad’s endemic area along the Chari River (Figure 4). Mali’s pattern of sporadic, dispersed infections of people and domestic animals also resembles Chad’s pattern of infected humans but with far fewer infected dogs. The current hypothesis is that most humans and animals in Mali in recent years probably also become infected by eating inadequately cooked aquatic animals such as fish that serve as transport or paratenic hosts of Guinea worm larvae.

The MGWEP’s main interventions for many years have included health education, distribution of cloth and pipe filters, promotion of safe drinking water sources, and containment of infected humans and animals. All seven villages that reported Guinea worm infections in 2020 had at least one source of safe drinking water, all received supplementary health education about thorough cooking of fish and proper disposal of fish guts, and distribution of cloth filters, and appropriate water sources in all were treated with Abate. On the basis of the new strategy’s effectiveness in Ethiopia and Chad, Mali’s GWEP discussed proactive tethering of dogs and cats with communities at risk in Segou and Mopti Regions before the transmission season began in 2021. Some communities agreed and began implementing the new approach in November, whereas others preferred instead to inspect their animals daily and tether any that showed signs of infection. Since September 2020, in a Peace-Health Initiative, Mali’s Ministry of Health, regional health leaders in Mopti, and local authorities in Tenenkou district of Mopti Region, which is an insecure district where dogs are bred and likely become infected, have fostered community-based discussions of peace, conflict, and health issues to help reduce insecurity. Assisted by The Carter Center and the nongovernmental organization HELP, the participating agencies have begun responding to priorities expressed by the communities, providing almost 40,000 cattle vaccinations, 300 cataract surgeries, Guinea worm education, water and sanitation needs, and rehabilitating a maternity ward. The MGWEP included 86 supervisory staff and 4,307 village volunteers in 2021.

Mali reported nine infected animals (eight dogs, one cat; six contained) in 2019; one human case (uncontained) and nine infected dogs (five contained) in 2020; and 17 infected animals (16 dogs, one cat) and two human cases in 2021. Mali contained 11 and identified the presumed source of 12 of its 19 Guinea worm infections in 2021. The impact of enhanced interventions in 2021 remains to be seen. Mali’s National Committee for Certification of Dracunculiasis Eradication, created by the minister of health in May 2015, met four times and made advocacy visits to Kayes, Koulikoro, and Segou Regions in 2019; met four times and convened advocacy workshops with veterinary services in Segou and Mopti Regions in 2020; and met once, conducted a workshop with veterinary services, and visited Sikasso Region in 2021.

### South Sudan.

The South Sudan GWEP (SSGWEP) reported 20,582 Guinea worm cases in 2006, its first year of autonomous operation following the Comprehensive Peace Agreement which ended Sudan’s long civil war in 2005 and led to South Sudan attaining full independence in 2011. South Sudan reported zero human Guinea worm cases for the first time in 2017 but discovered 10 cases in cattle camps in a newly pacified area the next year after 17 consecutive months of no reported cases. The SSGWEP reported four cases in 2019, one case in 2020, and four cases in 2021. It has reported only one infected animal ever, a dog in a household with human cases in 2015. As a measure of the SSGWEP’s surveillance system’s sensitivity, several submitted specimens that are not Guinea worms were diagnosed as *Sparganum*, a parasite with a similar life cycle, including an average of 7.2 spargana per year in 2017–2021 (all from human hosts). The program had 4,046 villages under active surveillance in 2017, 2,675 in 2019, and 851 in 2021. It also conducts integrated surveys for Guinea worm in cooperation with mass drug administration campaigns for onchocerciasis and trachoma, reaching almost 129,000 persons in 2019, more than 350,000 persons in 2020, and 919,257 persons in 2021. South Sudan increased its reward for reporting a human case from US$140 equivalent in 2017 ($23 for dogs) to US$400 in 2018, which declined to US$280 equivalent in 2020 ($26 for dogs) due to inflation. Spot checks of convenience samples found 71% to 75% of persons queried were aware of the reward for reporting humans in 2017–2020 and 83% in 2021, and 60% awareness of the animal reward in 2019. The SSGWEP investigated 25,182 rumors of Guinea worm infections in 2017, 65,997 rumors in 2019, and 48,589 rumors in 2021.

**Figure 5.**
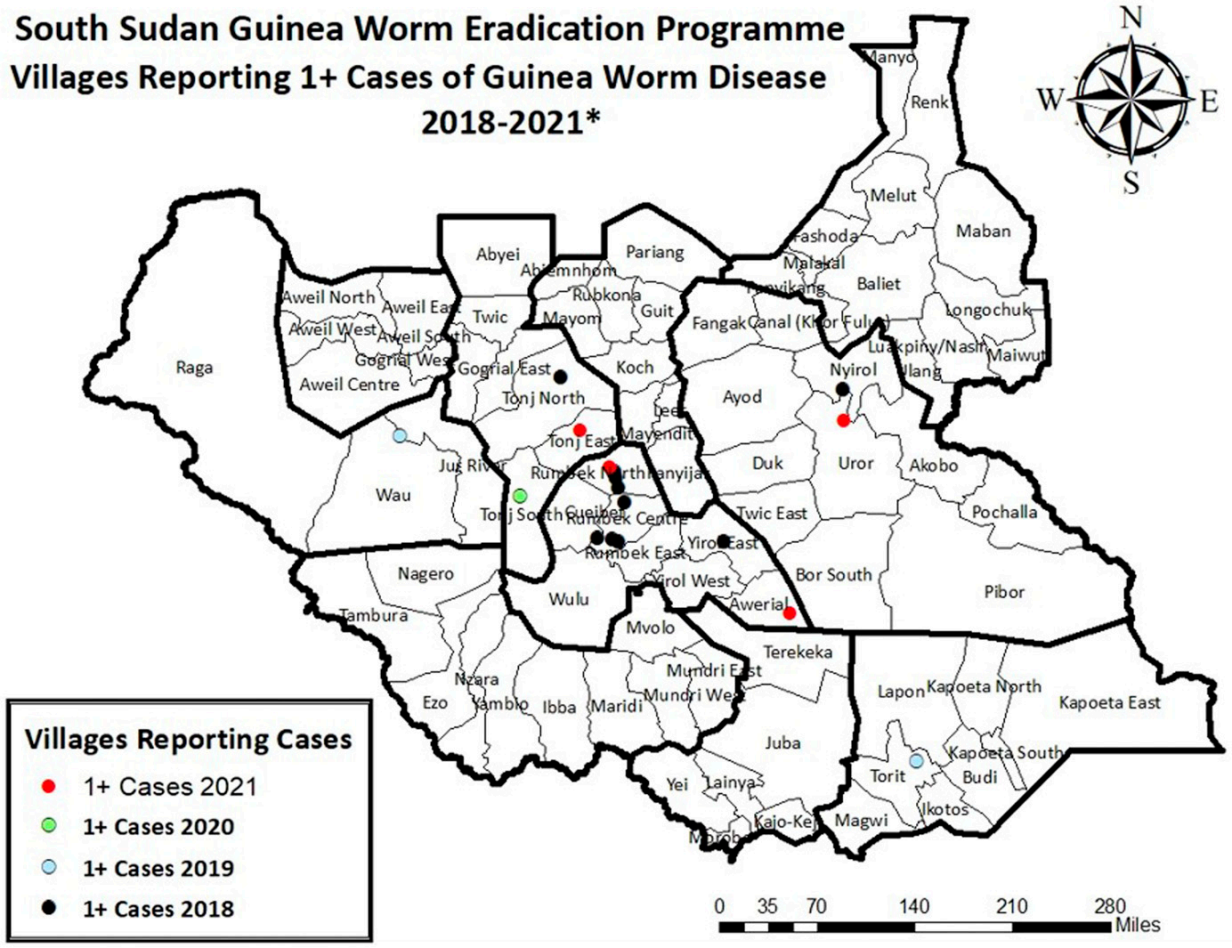
South Sudan Guinea Worm Eradication Program—villages reporting 1 or more cases of guinea worm disease, 2018–2021.

South Sudan’s 15 Guinea worm cases in 2018–2020 occurred in 12 localities (village or cattle camp); including three cases in one household in 2019 (Figure 5). Eight of the 15 cases were females; 11 were 15 years or older. Depending on patients’ age and gender, potential localities of cases here include home village, close and distant farms or gardens, different types of cattle camps, and areas in between, compared with only village and farm locations in most other endemic countries. One case, a male cattle keeper, illustrates the extreme mobility and challenges of tracing some South Sudanese patients: he walked more than 240 miles (400 km) and visited at least seven cattle camps in the 10 months before his worm emerged in July 2018.

With exceptionally strong political support and public health leadership since 2006, the SSGWEP covered more than 90% of its known endemic villages with health education, cloth and pipe filters, and Abate larvicide since before 2017. The proportion of currently endemic villages with at least one source of safe drinking water varied between 20% and 75% between 2018 and 2020, given the changes in affected localities. The program contained 10 of the cases (67%) reported in 2018–2020, and two of the four cases it reported from four villages in 2021 but could not identify a presumed source of infection for any. The SSGWEP apparently prevented forward transmission from the five known Guinea worm cases in 2019 and 2020 successfully by containing three (60%) of the cases and applying Abate larvicide within 2 weeks after the worms emerged in all three localities concerned. On the basis of the recent cases’ wide dispersal, the lack of a known source of infection, and no evidence of Guinea worm infection in animals, the hypothesis is that residual Guinea worm transmission in South Sudan may be due to a few chains of undetected human infections, abetted by sporadic insecurity and extreme mobility. Genetic analysis may help establish any otherwise inapparent linkages between Guinea worm infections.

The SSGWEP included 208 staff and 5,285 village volunteers in 2021. South Sudan established a National Committee for Documentation of Dracunculiasis Elimination in 2018. The committee met for the third time in 2019 and discussed a workplan for transitioning Guinea worm surveillance from the SSGWEP to primary care services. It did not meet in 2020 because of COVID-19 but reconvened in December 2021.

### Angola.

After the surprise discovery of a Guinea worm case for the first time ever in Angola in a girl with no history of foreign travel in 2018, Angolan authorities and the WHO investigated, searched nearby communities, and trained local health workers about the disease. They detected a second case and an infected dog in 2019 and a third case in 2020. All the infections were found in Cunene Province between January and April during the rainy season. None of the cases had traveled out of Angola. The WHO declared Angola officially endemic after the case in the third consecutive year.

Angola searched for Guinea worm infections in humans and animals in Cunene and five other provinces in 2019 during schistosomiasis mapping but found no additional infections. It publicized a reward equivalent to US$450 for reporting a case of Guinea worm disease by radio, television, and door-to-door; 38% of persons surveyed in areas at risk in 2019 were aware of the reward. A survey of approximately 1,000 persons in Cunene and two other provinces in 2019 found that 98.4% of persons queried denied having known about Guinea worm disease before, including 95% of persons in Cunene, and said there is no local name for it. Only 13% of villages in the three provinces had access to safe drinking water. The WHO helped train 1,400 health professionals in Guinea worm prevention that year, and The Carter Center signed a Memorandum of Understanding to also assist the Ministry of Health. The Carter Center and the WHO helped Angola train more health professionals virtually in 2020 and 2021, including some in how to use Abate, as well as community-based volunteers for 54 villages put under active surveillance with support provided by a WHO grant to Angola. The WHO assigned a full-time focal point for Guinea worm eradication and hired a data manager to assist in Angola. In 2021, Angola expanded surveillance to three adjacent provinces, had 61 villages under active surveillance, trained more than 200 health professionals and more than 1,000 community members, distributed cloth filters, conducted case searches in cooperation with malaria and other public health campaigns, and responded to 105 rumors. Angola has not reported a Guinea worm infection in a human or animal since March 2020.

The sources of Angola’s four infections are unknown. Genetic analysis has not yet established a clear link between the Guinea worms from Angola and any other country. Given what we know and do not know about Guinea worm’s occurrence in Angola—only three human cases and one infected dog found over 3 years, no prior history of Guinea worm infection in humans or animals ever, most people in the areas currently concerned say they do not know this disease and have no local name for it—the most plausible explanation may be that Angola’s outbreak began with one or more undetected case(s) imported into an area with unfavorable ecology.

### Global activities.

The WHO certified Kenya as free from dracunculiasis in 2018, bringing the total number of certified countries, areas, and territories to 199, with only Democratic Republic of Congo (DRC), Sudan, and five endemic countries (Angola, Chad, Ethiopia, Mali, South Sudan) not yet certified. The DRC and Sudan were expected to submit Country Reports to the International Commission for the Certification of Dracunculiasis Eradication (ICCDE) early in 2022 to begin their certification process. When WHO’s ICCDE met in Addis Ababa, Ethiopia in April 2019, it established a Working Group to draft supplemental criteria for certifying countries with sustained Guinea worm transmission in animals, and later confirmed that for the ICCDE to recommend certification, the latter countries needed to show evidence that they had halted Guinea worm transmission in animals as well as in humans. Deputy ICCDE chairman Dr. Joel Breman became ICCDE chairman when founding chairman Dr. Abdulrahman Al-Awadi died in 2019. Sadly, the campaign also lost three other major advocates for Guinea worm eradication, Former Malian President Amadou Toumani Toure and Former Ghanaian President Jerry John Rawlings in 2020, and Goodwill Ambassador Dr. Tebebe Yemane Berhan of Ethiopia in 2021.

The global campaign held joint reviews of the remaining endemic country programs and other countries remaining to be certified at The Carter Center in Atlanta each March in 2018–2019 and virtually in 2020 and 2021. Those international reviews were preceded by national reviews held separately in each country in December–January. During 2018–2021, the WHO Collaborating Center for Dracunculiasis Eradication at the CDC examined 638 specimens thought to be Guinea worms and submitted by countries. It confirmed 268 of them as *D. medinensis* by microscopic and/or molecular evaluation. In association with The Carter Center, the WHO Collaborating Center at the CDC continues to issue the newsletter of the global campaign, *Guinea Worm Wrap-Up*, almost monthly. Summaries of the status of the campaign are published annually in the CDC’s *Morbidity and Mortality Weekly Report*^7^ and in the WHO’s *Weekly Epidemiological Record*.^19^

In September 2018 the Bill & Melinda Gates Foundation and The Carter Center convened researchers and representatives from the Gates Foundation, The Carter Center, the WHO, the CDC, and the ministries of health of Ethiopia and South Sudan in Seattle, Washington, to review the status of the eradication campaign and related research efforts. The meeting discussed priorities for ongoing research, including developing a serological test to identify infected hosts, investigating small fish as possible transport hosts of the parasite, studying the impact of flubendazole on development of Guinea worm larvae, accelerating genomics analysis of worm specimens, and investigating how to link populations and surface water sources by using satellite data. Researchers from the Ethiopian Wildlife Conservation Authority, The Ethiopia Public Health Institute, The Carter Center, the Universities of Exeter and Roehampton, and others began collaborative ecological studies of baboons and dogs in the endemic area of that country in March 2018.

To encourage governments of endemic countries to support their national programs, the WHO and The Carter Center have engaged heads of state and ministers of health directly, convening ministers of health for an Informal Meeting to discuss Guinea worm eradication during annual World Health Assemblies (except in 2020 and 2021), including brief remarks by WHO Director-General Dr. Tedros Adhanom Ghebreyesus, inviting ministerial participation in annual Program Reviews, and publishing progress reports regularly in WHO’s *Weekly Epidemiological Record*, CDC’s *Morbidity and Mortality Weekly Report*, and CDC/Carter Center/WHO’s *Guinea Worm Wrap-Up*. WHO convened a cross-border meeting of GWEP leaders from Cameroon, Central African Republic, and Chad in August 2018, and facilitated bilateral cooperation to address transborder issues between Cameroon and Chad in 2019, 2020, and 2021, and between Angola and Namibia after the infection was discovered in Angola close to the Namibian border in 2018. WHO also led the international response when a suspected Guinea worm case later determined to be caused by a rare zoonotic *Dracunculus* of an unknown species, not *D. medinensis*, was reported in Vietnam in 2020.^20^

## DISCUSSION

The GWEP is addressing two main challenges to achieving eradication: animal infections in Chad, Ethiopia, and Mali and insecurity in Mali and South Sudan. Securing national political support and mitigating impacts of the COVID-19 pandemic are lesser obstacles. We now know that to achieve eradication, countries must stop *D. medinensis* infections in animals as well as in humans, and the ICCDE now requires convincing evidence that transmission is not occurring in animals or humans. Local veterinarians are working with the programs in all three countries with indigenous animal infections. Although recent sustained overall progress is encouraging, countries are intensifying interventions and research because we expect that reducing transmission will continue becoming harder and harder as infections decline and the most resistant endemic areas remain.

Mali has shown Guinea worm transmission can be sustained in an ecologically suitable endemic area, apparently by infections in dogs, for at least 4 years without known cases in humans. Mali is now introducing proactive tethering of dogs in parts of its endemic zone, following Ethiopia and Chad’s lead, as more communities agree to adopt that new strategy in addition to ongoing interventions. Mali’s Ministry of Health is leading efforts to address root causes of insecurity in some of the remaining endemic area, but to stop transmission among dogs, the program must escalate its engagement with members of endemic communities to encourage their cooperation with proactive tethering, safe disposal of fish entrails, and active surveillance of Guinea worm infections, despite the country’s current political difficulties.

The GWEPs of 15 of the 17 formerly endemic countries already certified as Guinea worm-free each had to address severe insecurity at some time in their work, including a civil war in Cote d’Ivoire and a large interethnic conflict in Ghana. South Sudan’s GWEP has faced civil war, political conflict, and ethnic disputes since before it gained independence in 2011, while insecurity became a severe constraint for Mali’s GWEP after a *coup d’etat* in 2012 rendered much of the country off-limits to outsiders. Health staff and village volunteers in GWEPs of both countries have persevered with courage and creativity, nonetheless, as shown by resilient measures of active surveillance. South Sudan has benefitted from very strong political support by the head of state, minister of health, other ministers, and other political leaders over the years. The SSGWEP’s dramatic achievements despite its unusually complex epidemiology are an especially impressive demonstration of a program’s ability to overcome serious security constraints. The widely dispersed Guinea worm infections in South Sudan with no traceable sources of infection by epidemiological investigation are cause for concern, despite effective containment of known cases and no known sustained animal infections. Adding routine genetic analysis of Guinea worms to ongoing active surveillance, case containment, and case investigation seems the best way forward in South Sudan as research in Chad has shown.^21^

With a ratio of more than 60:1 infected domestic dogs to human cases in 2018–2021, dogs have clearly been driving Guinea worm transmission in Chad, where the riverine endemic area resembles Mali’s, over the past decade, with incidental infection of humans. The similar 33% and 48% reductions in Guinea worm transmission to humans and dogs in Chad in 1 year (2021) may suggest parallel modes of transmission to humans and dogs by ingestion of infected transport hosts like fish rather than paratenic hosts such as frogs. Transmission via paratenic hosts might have prolonged infectivity and thus delayed impact of interventions beyond 1 year if humans or dogs were being infected mainly by consuming paratenic hosts. Steady expansion of Abate application and proactive tethering of almost all dogs in endemic areas during recent peak transmission seasons in Chad and Ethiopia have been followed by sharp reductions in Guinea worm infections of humans and animals in both countries within 1 year, despite the different ecologies involved. By blocking dogs’ exposure to contaminated water and/or fish as well as allowing prompt detection of dog infections and preventing contamination of water sources by dogs with emerging worms (tethering/containment of only known infected dogs prevents contamination by them, but not exposure of other dogs), proactive tethering is an effective enhanced strategy to help combat animal infections. As its dog infections decline, Chad’s program must double down on active surveillance, and on thorough investigation, tracing, and containment of each animal infection, while intensifying emphasis on proactive tethering, use of Abate, and safe water supply in endemic areas.

It is too soon to know whether proactive tethering of dogs and aggressive application of Abate in suspected endemic forested areas has suppressed transmission to humans, dogs, and baboons in Ethiopia. Sustained active surveillance, assiduous Abate application, and proactive tethering of dogs, as well as containment and tracing of human cases and dog infections, will be key in 2022. Provision of safe drinking water to unserved villages at risk and commercial farms is also needed urgently.

A broad coalition of epidemiologists, laboratory researchers, physicians, and veterinarians have helped explain some aspects of Guinea worm’s peculiar epidemiology in Chad, but not why animal infections apparently increased there so suddenly or why the parasite’s alternate life cycle via a paratenic and/or transport host did not manifest significantly, if at all, in other endemic countries earlier in the global campaign. Genomic profiling is increasingly helpful to reveal links among Guinea worm cases and animal infections and to help document the shrinking genetic diversity of worms in the end stage. In the elegant study of an outbreak in Chad, referenced earlier, genetic analysis not only confirmed the epidemiologic conclusion that this was a common-source outbreak, but also linked the 22 human cases with two canine infections. A serologic test to detect infection before the worm emerges and a tool to detect *D. medinensis* DNA in aquatic animals or water, if validated, would be useful innovations also, as would an effective anthelminthic to treat or prevent Guinea worm infections in dogs. All those potential new tools are being studied. Research to understand the nuances of transmission in three different ecological niches and to develop new tools must continue until the last Guinea worm is contained.

The surprise discovery of Guinea worm infections in Angola demonstrated the ability of the International Commission for the Certification of Dracunculiasis Eradication’s certification procedures to detect a wholly unexpected focus of infection in a presumed nonendemic country, but resolution of the unexpected focus in Angola requires continued surveillance, investigation, and vigilance.

The COVID-19 pandemic has had only modest impact on national GWEPs, which have continued to operate at 90% to 95% of pre-COVID-19 levels while taking measures to minimize risks to villagers and program staff from the virus. The pandemic did, however, delay some supervisory visits, research projects, and shipment of worm specimens for laboratory testing. The GWEPs’ lack of need to congregate large groups for mass drug administration or mass immunization campaigns has helped. GWEP staff have helped educate communities about prevention of COVID-19 and assisted distribution of associated COVID-19 materials.

Despite end-stage challenges, the eradication program reduced Guinea worm infections in humans globally by 51% between 2019 and 2020 and by 44% between 2020 and 2021, and it reduced infections in animals by 20% in 2019–2020 and by 45% between 2020 and 2021. The increasing numbers of rumors of Guinea worm cases and infections reported to national GWEPs provide assurance that recent declines in reported Guinea worm infections are not due to compromised surveillance.
